# Bariatric Surgery in Morbidly Obese Insulin Resistant Humans Normalises Insulin Signalling but Not Insulin-Stimulated Glucose Disposal

**DOI:** 10.1371/journal.pone.0120084

**Published:** 2015-04-13

**Authors:** Mimi Z. Chen, Claire A. Hudson, Emma E. Vincent, David A. R. de Berker, Margaret T. May, Ingeborg Hers, Colin M. Dayan, Robert C. Andrews, Jeremy M. Tavaré

**Affiliations:** 1 School of Clinical Sciences, University of Bristol, Bristol, United Kingdom; 2 School of Biochemistry, University of Bristol, Bristol, United Kingdom; 3 Bristol Dermatology Centre, Bristol Royal Infirmary, Bristol, United Kingdom; 4 School of Social and Community Medicine, University of Bristol, Bristol, United Kingdom; 5 School of Physiology and Pharmacology, University of Bristol, Bristol, United Kingdom; 6 Institute of Molecular and Experimental Medicine, Cardiff University School of Medicine, Cardiff, United Kingdom; John Hopkins University School of Medicine, UNITED STATES

## Abstract

**Aims:**

Weight-loss after bariatric surgery improves insulin sensitivity, but the underlying molecular mechanism is not clear. To ascertain the effect of bariatric surgery on insulin signalling, we examined glucose disposal and Akt activation in morbidly obese volunteers before and after Roux-en-Y gastric bypass surgery (RYGB), and compared this to lean volunteers.

**Materials and Methods:**

The hyperinsulinaemic euglycaemic clamp, at five infusion rates, was used to determine glucose disposal rates (GDR) in eight morbidly obese (body mass index, BMI=47.3±2.2 kg/m^2^) patients, before and after RYGB, and in eight lean volunteers (BMI=20.7±0.7 kg/m^2^). Biopsies of brachioradialis muscle, taken at fasting and insulin concentrations that induced half-maximal (GDR_50_) and maximal (GDR_100_) GDR in each subject, were used to examine the phosphorylation of Akt-Thr308, Akt-473, and pras40, *in vivo* biomarkers for Akt activity.

**Results:**

Pre-operatively, insulin-stimulated GDR was lower in the obese compared to the lean individuals (P<0.001). Weight-loss of 29.9±4 kg after surgery significantly improved GDR_50_ (P=0.004) but not GDR_100_ (P=0.3). These subjects still remained significantly more insulin resistant than the lean individuals (p<0.001). Weight loss increased insulin-stimulated skeletal muscle Akt-Thr308 and Akt-Ser473 phosphorylation, P=0.02 and P=0.03 respectively (MANCOVA), and Akt activity towards the substrate PRAS40 (P=0.003, MANCOVA), and in contrast to GDR, were fully normalised after the surgery (obese vs lean, P=0.6, P=0.35, P=0.46, respectively).

**Conclusions:**

Our data show that although Akt activity substantially improved after surgery, it did not lead to a full restoration of insulin-stimulated glucose disposal. This suggests that a major defect downstream of, or parallel to, Akt signalling remains after significant weight-loss.

## Introduction

Weight gain is a major risk factor for the development of Type 2 Diabetes (T2DM) and the relative risk increases dramatically with even a small increase in weight [1]. Weight gain is thought to have its effect by reducing the effectiveness of insulin action. Insulin-stimulated glucose uptake in skeletal muscle and adipocytes tissues is impaired in obesity and T2DM, causing peripheral insulin resistance (IR). Furthermore, insulin resistant obese individuals have reduced insulin sensitivity and maximal insulin-stimulated glucose uptake when compared to lean individuals [2, 3].

The molecular basis of IR is poorly understood. Insulin increases glucose uptake into muscle and adipose tissue by promoting the translocation of GLUT4 (glucose transporter isoform 4) from the cell interior to the plasma membrane [4, 5]. The fact that GLUT4 expression is not reduced in those with insulin resistance, such as in the obese and T2DM [6, 7], suggests that the major underlying defects in this condition are likely to lie in the insulin signal transduction pathways and/or the translocation of GLUT4 to the plasma membrane in skeletal muscle. Reduction in insulin stimulated phosphorylation of the insulin receptor and IRS-1, and reduced activity of PI3-kinase in T2DM suggest that defective insulin signalling may be, at least in part, responsible for insulin resistance [8].

Adipose tissue secretes a number of endocrine and inflammatory mediators, including free fatty acid (FFA) and adipokines (e.g. tumour necrosis factor α, Adiponectin, Leptin and Resistin) that influence insulin sensitivity and glucose homeostasis. Elevated FFA secretion associated with fat mass expansion is widely believed to play an important part in insulin resistance with it affecting the phosphorylation of IRS-1 and pathways that link into this [9, 10].

Akt is an insulin-stimulated serine/threonine-specific protein kinase that is well established as a critical component of the signalling mechanism used by insulin to stimulate glucose uptake [11–14]. A reduced ability of insulin to stimulate Akt in skeletal muscle has been observed in several studies of human IR [15–18], although others have disputed this [19–23]. This discrepancy could be due to the different experimental approaches used in these studies, including the use of different insulin infusion rates during hyperinsulinaemic euglycaemic clamps (HEC) or differences in the degree of obesity or glycaemic status of the subjects.

Studies that employ a single insulin concentration for both the control and affected group (i.e. obese, T2DM), either at the lower physiological or supra-physiological ends [15], provide limited insight into dynamic molecular activity. A given insulin concentration may fall outside the effective insulin range for one group and thus fail to provoke an observable physiological response, or only provoke a partial response, and thus lead to misleading results. Construction of a detailed insulin dose response curve specific to an individual is important in allowing dynamic assessment of the insulin signalling pathway and allows for better comparison between individuals [2].

Bariatric surgery can bring about substantial and sustainable weight loss in patients with morbid obesity, with patients losing 40–60% of their excess weight and maintaining this weight-loss for more than 10 years [24]. Despite this considerable weight-loss in patients with T2DM, studies have shown that although diabetes control is improved in the majority, a ‘cure’ (confirmed against 2009 ADA agreed criteria for remission [25]) is only seen in 40–50% of patients undergoing bariatric surgery [26, 27] and, of these, 10% see a recurrence in their diabetes each year [28]. One possible explanation for this is that insulin sensitivity is only partially normalised through augmentation of Akt activity in response to surgery.

In an attempt to clarify the role Akt plays in modulating insulin sensitivity after significant weight-loss through bariatric surgery, we carried out a detailed examination of the correlation between insulin-stimulated glucose disposal and Akt signalling in eight morbidly obese insulin resistant volunteers before and after a Roux-en-Y gastric bypass (RYGB). For each volunteer, we constructed a detailed insulin dose response curve for glucose disposal rates (GDR) calculated from HEC using 5 different insulin infusion rates. This was used to estimate the insulin concentrations required to induce half-maximal (GDR_50_) and maximal (GDR_100_) glucose disposal. In a second shorter clamp, forearm muscle biopsies were taken at three insulin infusion rates (fasting, GDR_50_ and GDR_100_), which allowed insulin signalling to be examined across the full physiological range for each individual. To study insulin signalling, we measured the state of phosphorylation of Akt on both Thr308 and Ser473, sites which play a role in the regulation of kinase activity and thus glucose uptake [29, 30]. We also assessed phosphorylation of the Akt substrate PRAS40, which we have shown previously to be an *in vivo* biomarker of Akt activity [30, 31]. Glucose disposal and insulin signalling were compared to eight lean volunteers.

## Materials and Methods

### Materials

Anti-phospho-Akt (Thr308), anti-phospho-Akt (Ser473) and anti-phospho-PRAS40 (T246) antibodies were purchased from Cell Signaling Technology (Danvers, MA, US) and an anti-total Akt2 antibody from Upstate (Hampshire, UK). Unless otherwise stated all other materials were purchased from Sigma Aldrich (Dorset, UK). Serum glucose samples were run using Abbot Architect c Systems reagent kit, with a limit of detection of 0.139mmol/L and an assay precision of <5% total CV. Serum insulin samples were run using Abbot Architect chemiluminescent microparticle immunoassay (CIMA), with a limit of detection of 1 μU/ml and an assay precision of <7% total CV.

### Research volunteers

Eight lean and eight obese white Caucasian volunteers participated in this study. The lean volunteers consisted of 4 females and 4 males, mean age 27.5±3.1 (mean ± SEM) years, BMI of 20.7±0.7 kg/m^2^ and were on no medication (Table 1). The obese volunteers consisted of 6 females and 2 males, mean age of 47.9±3.5 years and BMI of 47.3±2.2 kg/m^2^. Four of these obese volunteers had T2DM treated with oral medication (Table 2).

**Table 1:** Clinical characteristics and biochemical observations for lean volunteers.

Lean	Sex	Age (year)	Weight (kg)	Height (cm)	BMI (kg/m2)	Diabetes	Fasting BG (mmol/L)
1	m	28	65	188	18.4	no	4.6
2	f	29	54	160	21.1	no	5
3	f	47	53	165	19.5	no	4.9
4	m	25	65	181	19.8	no	5.1
5	f	22	62	178	18.9	no	5.1
6	m	21	75	189	21	no	5.2
7	m	29	86	186	24.9	no	4.7
8	f	19	60	165	22	no	4.1
**mean±SEM**		27.5±3.1	65.0±3.9	176.5±4.1	20.7±0.7		4.8±0.1

**Table 2:** Clinical characteristics and biochemical observations for obese volunteers before and after weight loss surgery.

ID	Sex	Age	DM	BMI (kg/m^2^)		Diabetes medication		Fasting glucose (mmol/L)		HbA1c (%)		Fasting free fatty acids (μmol/L)	
				Pre	Post	Pre	Post	Pre	Post	Pre	Post	Pre	Post
1	F	37	Yes	54.8	33.5	SU, TZD	None	12.3	5.2	7.5	5.9	528	470
2	M	45	Yes	50.3	38.3	Met, TZD	None	9.6	5.4	7.9	5.4	763	1143
3	M	57	Yes	37.4	29.5	Met, SU	None	11.9	6.2	8.5	7.4	978	489
4	F	53	Yes	54.5	46.7	Met, SU	None	8.6	6.3	7.8	7.1	861	866
5	F	56	No	44.4	36.2	None	None	4.6	4.1			771	838
6	F	56	No	48.2	41.1	None	None	6.1	5.1			923	1005
7	F	49	No	41.5	32.9	None	None	6	4			1500	604
8	F	30	No	47.4	35.6	None	None	3.6	4.7			845	653
Mean ± SEM		47.9±3.5		47.3±2.2	37±1.9*			7.8±1.2	5.1±0.3*	7.9±0.2	6.5±0.5*	896.1±98.6	758.5±86.3

Abbreviations for medications as follows; sulphonylureas (SU), metformin (Met) and rosiglitazone (TZD). HbA1c was only measured in patients with diabetes.

* = significant difference pre vs post with p<0.05

### Study design

All participants took part in a screening visit (visit 1), a long HEC (visit 2), a short HEC (visit 3) and a review visit (visit 4). For the obese participants, visits 2–4 were repeated at least 4 months (mean 6.8±1.2) post RYGB surgery. The RYGB procedures were performed laparoscopically by an experienced surgeon using an omega loop technique creating a 15 to 20 mL gastric pouch with a retrocolic antegastric Roux limb of 100 cm.

At visit 1 a medical history was taken and a physical examination performed. Blood samples were taken for full blood count, clotting screen, urea and electrolytes, liver function, and fasting free fatty acids (FFA) (measured by the colourmetrix method, Randox Laboratories Limited, UK).

### Ethics

Ethical approval was granted by South West 1 Research Ethics Committee and the study was sponsored by the University of Bristol, UK. Informed written consent was obtained from all the participants. All participants were adults older than 18 years. The study was carried out in accordance with the principles of the Declaration of Helsinki as revised in 2000.

### Long hyperinsulinaemic euglycaemic clamp for insulin dose response curve

Subjects attended at 8am having fasted from 11pm the night before and remained fasted throughout the test. All anti-diabetic medications were stopped for at least 24 hours before the clamp study. A cannula was placed in an antecubital vein for infusion of insulin (Actrapid, Novo Nordisk, UK) and glucose solution (5%, 10% or 20%; Baxter Pharmaceuticals, UK) and a second cannula placed retrogradely in a contralateral dorsal hand vein, the hand being kept in a heat pad for arterialised blood sampling.

Insulin was infused at five different rates, 0.2, 0.5, 1, 2 and 5 mU/kg/min and this was increased in a stepwise fashion every 2 hours [32]. A priming bolus dose of insulin was given at the beginning of each increase and blood glucose (BG) levels were monitored every 5 minutes using a glucose-monitoring device (One-Touch Ultra, Life Scan Limited, UK). Using this information, the rate of glucose infusion was adjusted to maintain BG at 5mmol/L. In addition to frequent bedside glucose monitoring, samples were obtained for laboratory serum blood glucose and insulin level [33].

Blood was immediately centrifuged, and the plasma was frozen and stored at −80°C until analysis. Steady-state glucose uptake was achieved at 90 minutes during each of the 2-hour infusion periods. GDR were calculated from infusion rates of glucose in the final 30 min of each rate of insulin. Insulin response curves were constructed using these data and subsequently used to determine the insulin infusion rates required to induce half-maximal (GDR50) and maximal (GDR100) glucose disposal for each individual.

### Short hyperinsulinaemic euglycaemic clamp

Participants re-attended for a short HEC within 6 weeks of the long clamp. Again they attended at 8am having fasted from 11pm the night before and remained fasted throughout the test. All anti-diabetic medications were stopped for at least 24 hours before the clamp study. Similar procedures were followed as described for the long hyperinsulinaemic euglycaemic clamp.

In brief, two cannulas were used: one was placed in the antecubital vein for infusion of insulin and glucose solution and the other retrograde in the cannula in a contralateral dorsal hand for blood sampling.

Insulin was infused at two different rates, the infusion rate predicted from the long clamp to achieve GDR_50_ and the infusion rate predicted to achieve GDR_100_. A priming bolus dose of insulin was given at the beginning of each increase and blood glucose (BG) levels were monitored every 5 minutes using a glucose-monitoring device (One-Touch Ultra, Life Scan Limited, UK). Using this information, the rate of glucose infusion was adjusted to maintain BG at 5mmol/L. In addition to frequent bedside glucose monitoring, samples were obtained for laboratory serum blood glucose and insulin level at the start and end of each infusion rate to ensure the expected insulin concentration had been obtained.

Muscle biopsies were taken at baseline, 45 minutes after the initiation of an insulin infusion rate that was predicted from the long clamp to achieve GDR_50_, and then again 45 minutes after the start of an insulin. Biopsies were briefly washed in ice-cold phosphate-buffered saline to remove residual blood and then immediately frozen in liquid nitrogen and stored at -80C until analysis.

An interval of 45 minutes was chosen as the original hyperinsulaemic clamp study by Defronzo RA [33] demonstrated that insulin concentrations reach a steady state by 20 minutes. In addition Middelbeek et al have shown that Akt Thr308 and AS160 phosphorylation was similar in biopsies taken at 30 minutes and 180 minutes into a hyperinsulaemic clamp study in lean and obese patients with and without diabetes [34].

### Muscle biopsies

Biopsies were carried out under local anaesthesia (10ml Bupivacaine, 0.5%) from the radial aspect of the non-dominant proximal forearm, which was chosen as it would allow us direct access to muscle with the least risk of infection in this particular group of patients. After an incision and blunt dissection, a 10 cm stretch of brachioradialis muscle was exposed for biopsy. Three 4 mm punch biopsies (30–50 mg of protein) were obtained at the proximal (first biopsy), distal (second biopsy) and middle (third biopsy) section of the exposed muscle with at least 5 cm between the biopsy sites. Between biopsies the wound was closed with steristrips and covered with gauze. At the end of the procedure the wound was closed using 4/0 Vicryl and 5/0 Ethilon.

### Tissue processing

Muscle biopsies were homogenised in 40 μl of ice-cold extraction buffer (50 mM Tris pH 7.5, 120 mM NaCl, 1% Nonidet P40, 40 mM β-glycerophosphate, 1 mM benzamidine, 1 mM EDTA, 50 mM NaF, 10 mM Na_4_P_2_O_7_, 10 μg/ml pepstatin, 10 μg/ml antipain, 10 μg/ml leupeptin, 2 μM microcystin, 5 mM Na_3_VO_4_, 2 mM phenylmethylsulfonyl fluoride) per mg of wet muscle using a Polytron homogenizer for a total of 1 minute. Tissue lysates were rotated for 45 minutes at 4°C before being cleared by centrifugation (16,000g for 15 minutes at 4°C). Protein concentration was determined using a commercial kit (protein assay from Bio-Rad, Hemel Hempstead, UK), and adjusted to 2 mg/ml with extraction buffer.

### Western blotting

50 μg aliquots of muscle protein were solubilised with 4x Laemmli sample buffer and, proteins were separated by SDS-PAGE using 4–12% gradient gels and transferred to polyvinylidene difluoride membranes (Millipore, Hertfordshire, UK). Membranes were blocked using 7.5% (w/v) non-fat dry milk in TBS-T (20 mM Tris pH 7.6, 137 mM NaCl, 0.1% Tween-20) for 1 hour. Membranes were washed, incubated with either anti-phospho-Akt (Ser473), anti-phospho-Akt (Thr308), anti-phospho-PRAS40 (T246), or anti-total Akt2 antibodies for 1 hour (all 1 μg/ml in TBS-T containing 5% w/v bovine serum albumin), before washing and incubating with the appropriate secondary antibodies diluted in TBS-T for 1 hour. Immunoblots were visualised using an Enhanced ChemiLuminescence detection system (ECL; GE Healthcare, Buckinghamshire, UK), and quantified by densitometry (Scion Image Software; Scion cooperation, Frederick, MD). Inter-blot comparison of phosphorylation levels was possible due to the inclusion of a positive control (lysate from insulin-stimulated rat adipocytes) on every SDS-PAGE gel to which other bands were normalised.

### Statistics

All data are expressed as the mean ± standard error of the mean (SEM). Clinical data between the obese and lean groups were compared using the unpaired *t*-test and between the obese patients before and after surgery using the paired *t*-test. When examining GDR, differences in insulin sensitivity between lean and obese groups were tested using multivariate analysis of covariance (MANCOVA). In this dose-response model, the initial GDR measurement and the repeated measures of GDR at different insulin infusion rates were taken into account as well as the pairing of subjects when comparing pre versus post operation GDR in the obese group.

Differences in trajectories of Akt-Ser473, Akt-Thr308 and PRAS40-T246 phosphorylation as a function of plasma insulin concentration between lean and obese groups were compared graphically and using the MANCOVA F-test. When comparing obese pre versus post operation Akt-Thr308, Akt-Ser473 and PRAS40-T246 phosphorylation, pairing of subjects was taken into account.

## Results

### Comparison of insulin sensitivity in the lean and obese subjects

**Fig 1:**
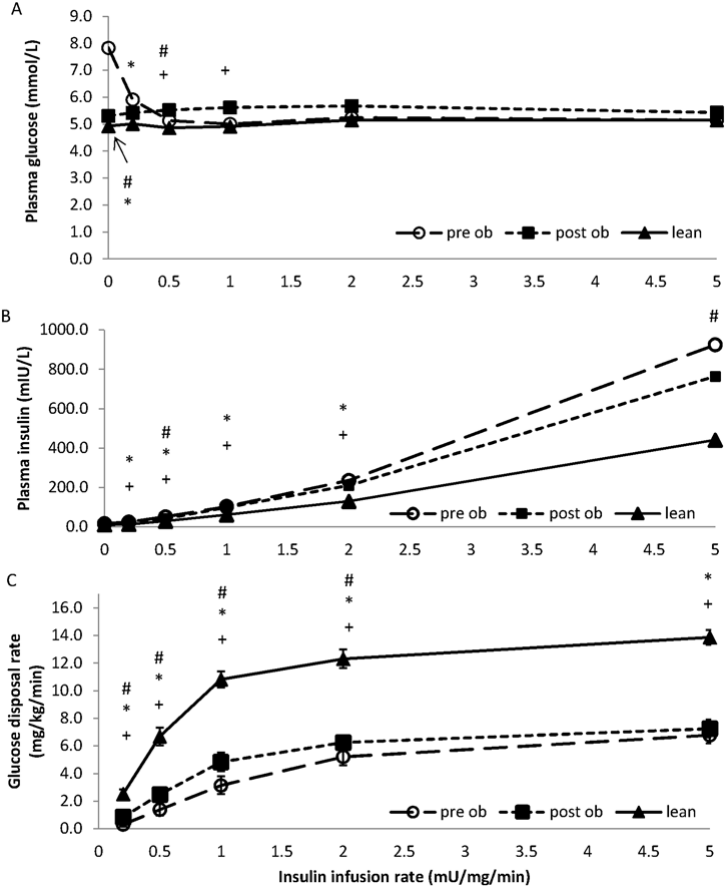
Glucose disposal rates in lean and obese volunteers before and after surgery. A comparison of glucose concentration (panel A), insulin concentrations (panel B) and glucose disposal (panel C), between lean (filled triangles) and obese volunteers before (open circles; pre-op) and after (filled squares; post-op) surgery, measured during a 10 hour hyperinsulinaemic euglycaemic clamp. Insulin infusion rates of 0, 0.5, 1, 2 and 5 mU kg-1 min-1 were used. Data points represent the mean ± SEM where * represents P<0.05 determined by unpaired t tests, between lean and pre-op obese volunteers; + represents P<0.05 determined by unpaired t tests between lean and post-op obese volunteers; # represents P<0.05 determined by paired t tests between pre-op and post-op obese volunteers. Glucose disposal rates were different between lean and obese volunteers (MANCOVA, P<0.001), and in obese volunteers before and after surgery (MANCOVA, P<0.05).

Fig 1 shows the glucose and insulin concentrations obtained from blood samples taken during the long HEC. Although the obese volunteers (pre-surgery) started the long clamp with a higher plasma glucose (7.8±1 vs 4.9±0.2 mmol/L, P = 0.007), glucose was subsequently maintained close to 5 mmol/L in both groups at all insulin infusion rates (Fig 1A). Plasma insulin concentrations were similar in each group at the start of the clamp but increased more in the obese subjects relative to the lean subjects (P = <0.01, Fig 1B).

**Fig 2:**
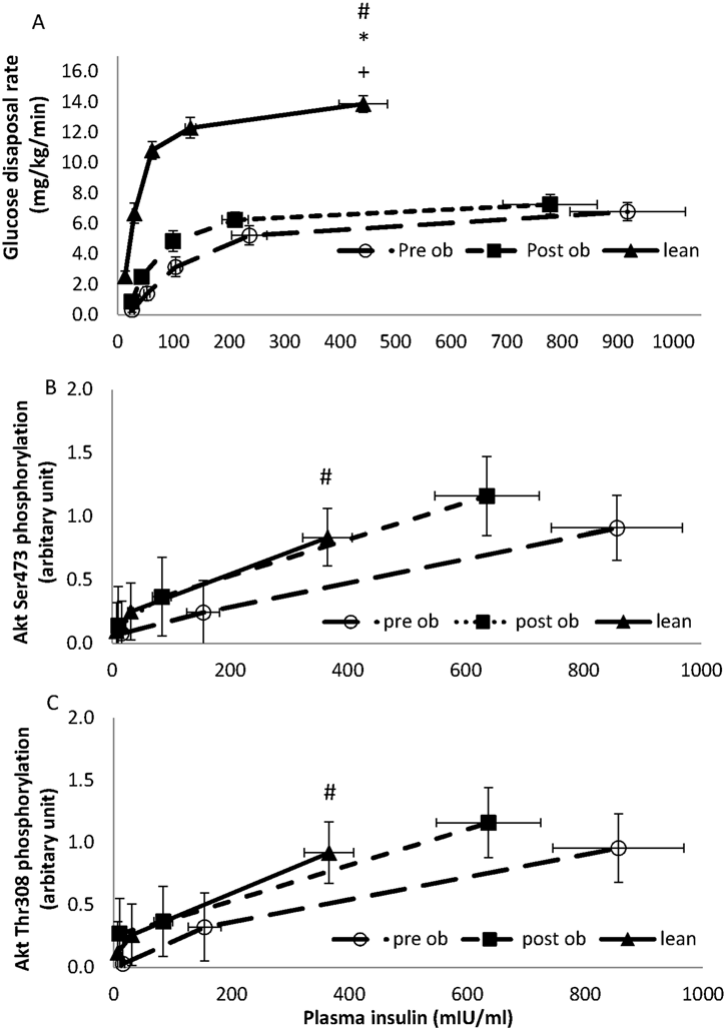
Summary of glucose disposal rates and insulin signalling in lean and obese volunteers, pre and post weight loss surgery. A comparison of glucose disposal (panel A), phosphorylation of Akt on Thr308 (panel B), phosphorylation of Akt on Ser473 (panel C) and phosphorylation of PRAS40 on T426 (panel D) between lean (filled triangles) and obese volunteers before (open circles; pre-op) and after (filled squares; post-op) surgery. The glucose disposal was measured during a 10 hour hyperinsulinaemic euglycaemic clamp with insulin infusion rates of 0.2, 0.5, 1, 2 and 5 mU kg-1 min-1. Data points represent the mean ± SEM with the glucose disposal plotted against the mean insulin concentration achieved at each insulin infusion rate.

The insulin dose response curve for glucose disposal in obese volunteers (pre-surgery) is significantly shifted down and to the right compared to lean subjects (Fig 1C). This is evidenced by lower GDR at all insulin infusion rates examined and higher insulin infusion rate or plasma insulin concentration required to induce GDR_50_ (MANCOVA P*<*0.001). This reduction could be readily seen whether we plotted GDR against insulin infusion rate (Fig 1C) or against the plasma insulin concentration achieved at each infusion rate in each subject (Fig 2A).

Phosphorylation of Akt on Thr308 (B) or Ser473 (C), and PRAS40 on T426 (D) were determined by western blotting for each volunteer and quantified by densitometry using an internal standard. Data points represent the mean ± SEM of the phosphorylation score against the mean insulin concentration seen at baseline, GDR_50_ and GDR_100_. Before surgery, Akt phosphorylation on Thr308 across the dose response profile was reduced in the obese volunteers when compared to the lean (Fig 2B; P = 0.06 using a MANCOVA F-test); Ser473 phosphorylation also showed a tendency to a reduction (Fig 2C; P = 0.15). Weight loss increased skeletal muscle Akt phosphorylation on both Thr308 and Ser473 (P = 0.020 and P = 0.027, respectively; MANCOVA F-test). PRAS40 was reduced in the obese compared with lean volunteers across dose response profile (P = 0.006, using MANCOVA F-test). After surgery, PRAS40 phosphorylation on T246 recovered in the obese volunteer (P = 0.003, MANCOVA F-test), and was no longer significantly different from the lean controls (P = 0.46, respectively; MANCOVA F-test).

Glucose disposal rates were different between lean and obese volunteers (MANCOVA, P<0.001), and in obese volunteers before and after surgery (MANCOVA, P<0.05). Phosphorylation of Akt on Thr308 (C) and Ser473 (B) were compared between lean and obese volunteers (P = 0.06 and P = 0.15 respectively), and in obese volunteers before and after surgery (P = 0.020 and P = 0.027 respectively). However, there were no statistical differences between the lean and obese volunteers after surgery.

### Comparison of insulin-stimulated Akt and PRAS40 phosphorylation between lean and obese subjects

Insulin signalling in obese versus lean volunteers were compared. Before surgery, Akt phosphorylation on Thr308 across the dose response profile was reduced in the obese volunteers when compared to the lean non-insulin resistant volunteers (Fig 2B; P = 0.06 using a MANCOVA F-test). Ser473 phosphorylation also showed a tendency to a reduction (Fig 2C; P = 0.15). PRAS40 phosphorylation was also reduced in the obese compared with lean volunteers across the dose response profile (Fig 2D, p = 0.006, using a MANCOVA F-test).

### Comparison of insulin sensitivity in obese subjects before and after surgery

Surgery resulted in a mean weight-loss of 29.9±4.0 kg (135.2±7.1 vs 105.3±6.9 kg, P<0.001, Table 2), with accompanied glycaemic improvement. In those volunteers with diabetes, reductions in fasting plasma glucose (7.8±1.2 vs 5.1±0.3mmol/L, P = 0.01) and HbA1c were observed (7.9±0.2 vs 6.5±0.5%, P = 0.02), together with cessation of all diabetes medications (Table 2).

The plasma insulin concentrations attained were similar across all infusion rates, except during the 0.5 and 5 mU/kg/min infusion rates where the insulin concentrations achieved were lower post-operatively (42.7±4.5mIU/L vs 52.3±5.5 mIU/L, P = 0.03; and 765±96.2 mIU/L vs 925±121.6 mIU/L, P = 0.04; respectively, Fig 1B).

Weight loss resulted in a significant improvement in insulin-stimulated glucose disposal (MANCOVA, P = 0.03, Figs 1C and 2A) with the response curve shifting to the left (i.e. less insulin was required to induce a GDR_50_ response (73.3±12.8 post-surgery vs 131.3±25.7 mIU/L pre-surgery, P<0.001), Fig 2A). Despite this improvement, the obese group still remained more insulin resistant than the lean (i.e. obese patients required more insulin to induce a GDR_50_ response than the lean (73.3±12.8 post-surgery vs 33.8±11.9 mIU/L lean, P = 0.012, Fig 2A); at an insulin infusion rate of 1 mU/kg/min, glucose disposal was 4.8±0.7 mg/kg/min in obese volunteers post-surgery versus 10.8±0.6 mg/kg/min in the lean (P<0.001). Maximal GDRs reached 7.3±0.6 mg/kg/min in the obese post-surgery versus 13.9±0.5 mg/kg/min in the lean (P<0.001).

### Comparison in insulin-stimulated Akt and PRAS40 phosphorylation in obese subjects before and after surgery

**Fig 3:**
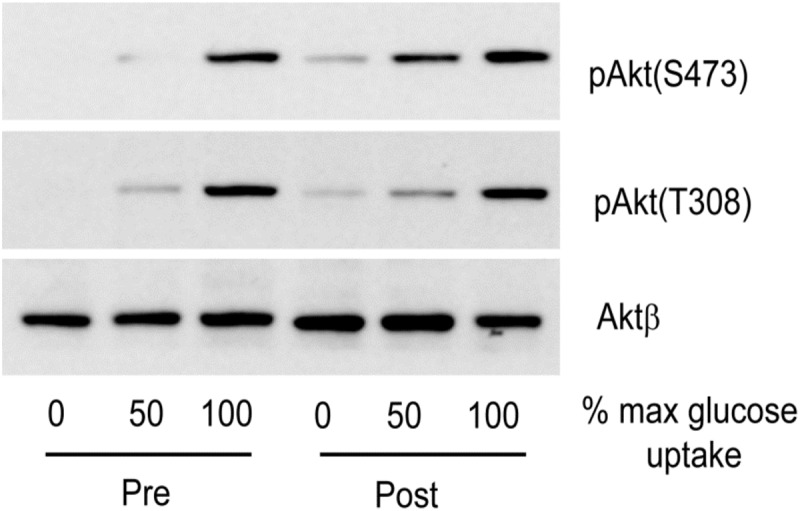
Insulin signalling in obese volunteers, pre and post weight loss surgery. Obese volunteers (pre and post-op) underwent a 3-hour euglycaemic hyperinsulinaemic clamp and muscle biopsies were taken at fasting (0) and insulin infusion rates predicted to achieve half maximal (GDR_50_) and maximal (GDR_100_) rates of glucose disposal. The extent of phosphorylation of Akt on Thr308, Ser473 and PRAS40 on T246 was determined by western blotting. The results shown are from a single individual, but are representative of the eight obese volunteers [see Materials and Methods].

As shown in Fig 2B and 2C, weight loss was accompanied by an increased skeletal muscle Akt phosphorylation on both Thr308 and Ser473 (P = 0.020 and P = 0.027, respectively; MANCOVA F-test). PRAS40 phosphorylation on T246 also improved (P = 0.003, MANCOVA F-test). After surgery insulin-stimulated Akt-Thr308 and Ser473 phosphorylation, as well as PRAS40-Thr246 phosphorylation were not significantly different from the lean controls (P = 0.6, P = 0.35 and P = 0.46, respectively; MANCOVA F-test). A representative experiment is shown in Fig 3.

## Discussion

This study aimed to explore the molecular basis underlying the impairment in insulin stimulated glucose disposal observed in obesity. A comprehensive examination of Akt phosphorylation was performed in skeletal muscle of lean and morbidly obese individuals at fasting and at higher plasma insulin concentrations, corresponding to half maximal (GDR_50_) and maximal (GDR_100_) levels of glucose disposal for each individual. Repeating these studies four months after the obese participants underwent a Roux-en-Y gastric bypass, a procedure that led to significant weight loss and cessation of requirement for diabetes medication, enabled us to look at the effect of weight loss on Akt phosphorylation and activity, and glucose disposal rates in response to insulin. We used lean controls as a benchmark for optimum insulin signalling and sensitivity.

As expected, when examining whole body glucose disposal, the morbidly obese individuals were significantly less insulin sensitive than the lean volunteers. The insulin dose response curve was shifted down and to the right. GDRs were lower at all insulin infusion rates examined (Figs 1C and 2A). RYGB surgery-induced weight loss (22.9±4.0kg) significantly improved insulin-stimulated glucose disposal with the greatest change occurring at GDR_50_, and no change at GDR_100_. While the glucose disposal profile still remained insulin resistant relative to lean volunteers, the improvement was clinically meaningful as it was accompanied by normalisation of fasting plasma glucose and HbA1c, and cessation of diabetes medication in all four of the volunteers with diabetes (Table 2).

Previous studies that have examined the effect of bariatric surgery on IR have used either a measure of basal insulin sensitivity, HOMA, or a HEC at a single insulin infusion rate equivalent to 1mU/kg /min. In these studies, HOMA and glucose disposal improved significantly [35–38]. The improvement we see at 1mU/kg/min is of similar magnitude to that reported in other studies [37], however our study is the first to undertake a detailed examination across a range of plasma insulin concentrations.

Glucose disposal in the obese subjects after surgery remained highly insulin resistant when compared with the lean subjects despite significant weight loss, improved glycaemic control and metabolic parameters. In contrast, surgery-induced weight loss improved Akt-Thr308 phosphorylation (pre vs post surgery, P = 0.020; MANCOVA F-test), and the phosphorylation of the downstream Akt substrate PRAS40 on Thr246 (P = 0.003, MANCOVA F-test), such that they became indistinguishable across the insulin dose response profile to those observed in lean subjects (P = 0.6 and 0.46, respectively; MANCOVA F-test). In human biopsy samples and human platelets we have previously shown that Thr308 is an excellent biomarker for Akt kinase activity, which is consistent with this observation [30, 31]. Taken together, this suggests that the reduced insulin-stimulated Akt-Thr308 phosphorylation, and activity of this kinase towards downstream substrates such as PRAS40, as observed in obese patients before surgery when compared to lean subjects (P = 0.06 and P = 0.006 for Akt-Thr308 and PRAS40-Thr246, respectively; MANCOVA F-test), may reflect a state of ‘Akt- resistance’ to insulin action in the obese, a reversible phenomenon that can be improved by surgery induced weight loss.

We also saw an improvement in insulin-stimulated Akt-Ser473 phosphorylation after surgery in obese subjects (pre vs post surgery, P = 0.027; MANCOVA F-test). However, the reduction in insulin-stimulated Akt-Ser473 phosphorylation in obese subjects before surgery, when compared to lean individuals, was not as obvious as it was for Thr308 phosphorylation and would require a larger sample size to confirm that it normalises in the same manner as Akt-Thr308 phosphorylation after weight loss.

These data suggest that weight loss can lead to an improvement in insulin signalling, which is accompanied by a small but clinically significant improvement in GDR. Therefore, any intervention designed to improve insulin signalling in obesity-induced IR could enhance glucose disposal in a clinically meaningful manner. The data further suggest that a major molecular defect in glucose disposal downstream from Akt signalling, or parallel to it, remains after surgery. The expression of the insulin responsive glucose transporter GLUT4 in muscle has been reported not to change after gastric bypass surgery [36]. Alternative explanations for this remaining defect in glucose disposal could, therefore arise from: (i) a mis-localisation of GLUT4 in the cells as previously proposed by Garvey et al [39], (ii) an inability of Akt and/or its substrates to couple to the GLUT4 translocation machinery, or (iii) a defect in a parallel Akt-independent signalling pathway such as the Cbl-CAP-C3G pathway [40] or Munc18c tyrosine phosphorylation [41]. The contribution of these mechanisms to the continuing insulin resistance observed requires further detailed investigation.

The mechanism by which weight loss leads to the substantial improvement in Akt-Thr308 and Akt activity leading to PRAS40-Thr246 phosphorylation is unknown. Circulating FFA levels were higher in the obese subjects prior to surgery when compared to lean individuals (759±86 μmol/L versus 561±80.8 μmol/L, respectively; P = 0.06, unpaired student *t*-test). Elevated FFA have been reported to reduce insulin-stimulated IRS1 tyrosine phosphorylation, possibly via activation of PKCθ, which would be expected to reduce insulin-stimulated Akt activation [42]. This does not appear to play a role in the current study as no change in circulating FFA were observed in the obese subjects after surgery (Table 2), although we cannot exclude the possibility that intramyocellular levels of FFA were reduced after surgery so reducing local PKCθ activity.

RYGB surgery increases GLP-1 production immediately after the operation and remains elevated for at least 18 months [43, 44]. Studies have shown that GLP-1 activates Akt signalling, either via transactivation of the epidermal growth factor receptor or induction of cAMP and cAMP response element-binding dependent transcription of IRS2 [45]. Thus the increase in GLP-1 could be one way that bypass surgery improves insulin signaling. This may partially explain the normalisation of Akt activity after RYGB, however, there is no direct evidence linking increased GLP-1 production and improved phosphorylation of Akt in the morbidly obese individuals undergoing bariatric surgery.

In previous studies, two hour insulin infusion rates were employed before biopsies were taken as it was considered that this time interval was required before insulin concentrations and glucose disposal become stable [46]. However Defronzo’s original hyperinsulinaemic study showed that insulin concentration reach a steady state after 20 minutes [33] and Middelbeek et al have shown that Akt Thr308 and AS160 phosphorylation was similar in biopsies taken at 30 minutes and 180 minutes into a hyperinsulinaemic clamp study in lean and obese patients with and without diabetes [34]. In light of this we elected to do our muscle biopsies after only 45minutes of insulin infusion. In addition looking at changes with a shorter insulin infusion time is more physiological as high insulin concentrations are rarely elevated for 2 hours in *vivo*. It may well be that if we had biopsied after 2hours of insulin we may have seen slightly different levels of phosphorylation of AKT [47].

It remains to be established whether the glucose disposal profile of our obese volunteers would approach that of lean individuals upon further weight reduction, as this might be expected to reduce circulating FFA levels to normal [48, 49]. The findings that only 40–50% of patients with diabetes see a remission in diabetes after RYGB, and of those 10% see a reoccurrence each year despite an improvement in insulin secretion and insulin receptor concentration, suggest that this does not occur. However in 30 RYGB patients we have seen further improvements in IR between 6 and 18 months with only 5% more weight loss [50].

To conclude, our data suggest that an improvement in Akt-Thr308 phosphorylation and its kinase activity induced by weight loss brought about by bariatric surgery is associated with a small but clinically significant improvement in glucose homeostasis. However a major defect in glucose disposal, most likely in muscle, still remains, which underlies IR in morbid obesity and which is downstream or parallel to Akt signalling. This defect may be overcome once further weight loss has been attained and circulating FFA levels are normalised, or may remain and explain why resolution of diabetes after bariatric surgery is not seen in all cases and is not always permanent.
